# 

**Published:** 1912-03

**Authors:** 


*THE BRISTOL MEDICO-CHIRURGICAL SOCIETY.*

LIBRARY OF CONGRESS.
MAR 29 1912
SMITHSONIAN DEPOSIT.

*Editor:* R. SHINGLETON SMITH, M.D., B.Sc.

WITH WHOM ARE ASSOCIATED

J. MICHELL CLARKE, M.A., M.D.,

J. LACY FIRTH, M.S., M.D., JAMES SWAIN, M.S., M.D.

P. WATSON-WILLIAMS, M.D., *Assistant Editor.*

*Editorial Secretary:* J. A. NIXON, B.A., M.B.

### CONTENTS.

| | Page |
|---|---|
| Lister: An Appreciation by Robert Roxburgh, M.B., F.R.C.S. Ed. ... | 1 |
| Direct Laryngoscopy, Bronchoscopy and Œsophagoscopy. By P. Watson-Williams, M.D. Lond. ... | 11 |
| A Case of Chronic Interstitial Pancreatitis of Uncertain Origin. By J. Michell Clarke, M.A., M.D. Cantab., F.R.C.P. ... | 25 |
| Operations on the Uterus and Appendages during Pregnancy. By W. C. Swayne, M.D. Lond. ... | 31 |
| Some Cases illustrating the Difficulty and Importance of Early Diagnosis in Acute Intestinal Obstruction. By Ernest W. Hey Groves, M.S., F.R.C.S. ... | 37 |
| Some Observations on Tuberculosis of the Nose and Pharynx. By A. J. Wright, M.B., F.R.C.S. ... | 45 |
| Some Gall-Bladder Cases presenting Features of Interest. By E. H. E. Stack, M.B., B.C., F.R.C.S. ... | 49 |
| The End-Results of Forty-one Operations for Internal Derangements of the Knee-Joint. By A. Rendle Short, M.D., B.S., B.Sc. Lond., F.R.C.S. ... | 52 |
| Progress of the Medical Sciences: | |
| Medicine. By Carey Coombs, M.D. Lond. ... | 64 |
| Surgery. By E. W. Hey Groves, M.D., M.S. Lond. ... | 68 |
| Obstetrics. By D. C. Rayner ... | 72 |
| Reviews of Books ... | 74 |
| Editorial Note ... | 84 |
| Notes on Preparations for the Sick ... | 84 |
| The Library of the Bristol Medico-Chirurgical Society ... | 87 |
| Meetings of Societies ... | 89 |
| Obituary Notice ... | 93 |
| Local Medical Notes ... | 94 |

